# Determinants of antenatal depression and postnatal depression in Australia

**DOI:** 10.1186/s12888-018-1598-x

**Published:** 2018-02-20

**Authors:** Felix Akpojene Ogbo, John Eastwood, Alexandra Hendry, Bin Jalaludin, Kingsley E. Agho, Bryanne Barnett, Andrew Page

**Affiliations:** 1 Translational Health Research Institute, School of Medicine, Western Sydney University, Campbelltown Campus, Locked Bag 1797, Penrith, NSW 2571 Australia; 2 Ingham Institute for Applied Medical Research, University of New South Wales, 1 Campbell Street, Liverpool, NSW 2170 Australia; 3 School of Women’s and Children’s Health, University of New South Wales, Kensington, Sydney, NSW 2052 Australia; 4 Menzies Centre for Health Policy, Charles Perkins Centre, School of Public Health, Sydney University, Sydney, NSW 2006 Australia; 5 School of Public Health, Griffith University, Gold Coast, QLD 4222 Australia; 6 Department of Community Paediatrics, Sydney Local Health District, Croydon Community Health Centre, 24 Liverpool Rd, Croydon, NSW 2132 Australia; 7 National Centre for Immunisation Research and Surveillance, The Children’s Hospital at Westmead, Locked Bag 4001, Westmead, NSW 2145 Australia; 8 Healthy People and Places Unit, South Western Sydney Local Health District, Liverpool, NSW Australia; 9 St John of God Health Care, Blacktown, NSW Australia

**Keywords:** determinants, antenatal, depression, perinatal, postnatal, Australia, pregnancy

## Abstract

**Background:**

Depression is a leading source of morbidity and health loss in Australian women. This study investigates the determinants of antenatal depressive symptoms and postnatal depressive symptoms in an Australian population, including people from culturally and linguistically diverse (CALD) backgrounds.

**Method:**

The study used a retrospective cohort of mothers of all live births in public health facilities in 2014 (*N* = 17,564) within South Western Sydney Local Health District and Sydney Local Health District in New South Wales, Australia. Prevalence of antenatal and postnatal depressive symptoms were estimated for the cohort. Multivariate logistic regression models were conducted to investigate the sociodemographic, psychological and health service determinants of antenatal and postnatal depressive symptoms, measured using the Edinburgh Postnatal Depression Scale (EPDS).

**Results:**

The prevalence of antenatal and postnatal depressive symptoms was 6.2% and 3.3% of the cohort, respectively. Significant risk factors for maternal depressive symptoms during pregnancy were, a lack of partner support, history of intimate partner violence, being from the CALD population and low socioeconomic status. Self-reported antenatal depressive symptoms were strongly associated with postnatal depressive symptoms. Risk factors for postnatal depressive symptoms were similar to those for antenatal depressive symptoms, as well as assisted delivery.

**Conclusion:**

Factors relating to demographic and psychosocial disadvantage were associated with subsequent antenatal and postnatal depressive symptoms in New South Wales, Australia. Our study suggests that screening for probable depression and timely referral for expert assessment of at-risk mothers may be an effective strategy to improve maternal mental health outcomes.

## Background

Depression is a major public health issue worldwide and was among the leading source of health loss in terms of disability-adjusted life years among Australian women of reproductive age in 2016 [1, 2]. Maternal depression during pregnancy (antenatal depression) and postnatal depression have both short- and long-term consequences for the mother, the child and family. For the infant, these may include low birth weight, psychological issues, increased risk of experiencing diarrhoea and suboptimal infant feeding patterns [3–6]. Lower rates of immunisation, and reduced growth and developmental trajectories have also been reported in infants whose mothers had perinatal depression [7]. In addition, stress, poor social interaction and lost productivity have been documented in pregnant women with depression [8, 9].

In Australia, recent studies have indicated that maternal depression during pregnancy is associated with adverse perinatal outcomes [10, 11]. Other Australian studies have reported higher prevalence for antenatal depression, from 6% [4, 10] to 17% [12] and postnatal depression, from 6% to 12% [4, 12, 13]. A study conducted among women living in a socioeconomically disadvantaged community in South Australia indicated that the incidence of antenatal depressive symptoms over a seven-month period was 30%, reflecting variation in the burden of perinatal depression in women across the socioeconomic scale [14].

Previous studies conducted in Australia have shown a range of factors associated with antenatal and postnatal depression. Risk factors for antenatal depression include low self-esteem, anxiety, low social support, negative cognitive style, major life events, low income and history of abuse [12, 14], domestic violence during pregnancy and having recently hit someone else in anger [14]. Risk factors for postnatal depression include antenatal depression and history of depression [12], poor health status of the mother, not breastfeeding, difficult financial situation and being born overseas [13]. Furthermore, analysis of data from the beyondblue National Depression Program in Australia found that antenatal depression, a prior history of depression and limited partner support were the strongest risk factors for postnatal depression [15].

The Edinburgh Postnatal Depression Scale (EPDS) is routinely used for pregnant women in many Australian primary health care settings to identify and refer for expert assessment and management, women who may be experiencing or are at risk of perinatal depression. The National Postnatal Depression Program recommends the EPDS as a screening device for perinatal depression, and advocates for ongoing research in perinatal depression to inform policy formulation, and targeted and evidence-based initiatives [4, 16]. Accordingly, the present study aimed to investigate socioeconomic, psychosocial and health service determinants of maternal depressive symptoms during pregnancy and postnatal depressive symptoms in Australia, including people from culturally and linguistically diverse (CALD) populations.

## Methods

### Data source

The study was based on a retrospective cohort of mothers of all live births (*N* = 17,564) in public health facilities within South Western Sydney Local Health District (SWSLHD) and the Sydney Local Health District (SLHD) in 2014, in the state of New South Wales (NSW), Australia. Antenatal data, routinely collected by qualified midwives, were linked to routinely collected postnatal data (by skilled community health nurses), using individual identifiers. These data were stored in the Information Management & Technology Division (IM&TD) database of the health districts. After ethics approvals were obtained, the data were cleaned and coded for analysis.

### Study setting

SWSLHD and SLHD represent approximately 52 % of the Sydney metropolitan region, with a population of over 1,457,100 people. This region of Sydney has one of Australia’s most culturally diverse populations [17, 18], with approximately 50 % of mothers born overseas, with mothers from Middle Eastern countries (10%), South East Asia (8%) and Southern Asia (8%) most commonly represented [19]. The health districts provide health services to a socioeconomically diverse population, with some geographic areas in South Western Sydney representing some of the most socioeconomically disadvantaged and advantaged areas in the Sydney metropolitan region [10].

### Risk factors

Study variables included: maternal age (categorised as less than 20 years, 20–34 years or over 35 years); area-based socioeconomic status (SES, categorised as low, medium or high); partner support (categorised as Yes or No/unsure); culturally and linguistically diverse (CALD, categorised as Yes or No); history of physical intimate partner violence (IPV, categorised as Yes or No); fear of partner/ex-partner (categorised as Yes or No); history of pre-existing antenatal health problems (such as diabetes mellitus and hypertension, categorised as Yes or No); and type of delivery (categorised as normal vaginal, assisted vaginal or caesarean delivery). Sociodemographic, psychological and health service data were collected in the first prenatal clinic visit for the index pregnancy, and information on the type of delivery was collected soon after birth.

Area-based SES was measured using the Socio-Economic Index for Areas (SEIFA), based on the mother’s address. SEIFA is a measurement indicator developed by the Australian Bureau of Statistics that ranks areas in Australia according to relative socio-economic advantage and disadvantage [20]. Deciles of SES were categorised into high (top 10% of the population), medium (middle 80% of the population) and low (bottom 10% of the population) groups, consistent with previous publications [10, 19]. CALD is a term used for communities with diverse language, ethnic background, nationality, dress, traditions, food, societal structures, art and religion characteristics [21]. IPV information was collected during antenatal period based on the NSW Routine Domestic Violence Screening policy, where mothers were asked: “Within the last 12 months, have you been hit, slapped or hurt in other ways by your partner or ex-partner (physical IPV)?” or “Are you frightened of your partner or ex-partner (psychological IPV)?”

### Outcome variables

In the present study, maternal depressive symptoms during pregnancy and postnatal depressive symptoms were the main outcomes, measured using the EPDS, which has been validated in the antenatal and postnatal periods [22–24]. The EPDS has been recommended as a screening tool for perinatal depression among women in Australia [4]. Midwives collected information on sociodemographic and psychosocial characteristics, and maternal depressive symptoms during pregnancy at the first antenatal care visit. For non-English speaking mothers, the English version of the EPDS was administered through qualified interpreters, certified by the National Accreditation Authority for Translators and Interpreters in Australia. The overall number of antenatal depressive symptoms was calculated to achieve a total score (out of 30) and was then coded as a categorical variable (score < 13 or score ≥ 13), with a score of greater or equal to 13 suggestive of antenatal depressive symptoms [24].

Data on postnatal depressive symptoms were collected within the first six weeks of birth. The total number of postnatal depressive symptoms was also tallied to obtain a total score (out of 30) and was then coded as a categorical variable (score < 13 or score ≥ 13) to indicate scores suggestive of postnatal depressive symptoms [24]. In the present analysis, the EPDS cut-points used to indicate probable depression were selected based on previously published studies [10, 13, 25, 26] and the NSW government guidelines on improving mental health outcomes for parents and infants [27].

The EPDS is the most common and acceptable screening tool for identifying depressive symptoms in the perinatal period worldwide, with a reported sensitivity of 68–86% and specificity of 78–96% [23, 24]. In Australia, a study that used the EPDS among a sample of 4148 women, reported a sensitivity of 100% and specificity of 89% [28]. Studies have reported a positive predictive value (PPV) for clinical depression [23] and a PPV of approximately 70% with an EPDS score of > 12 [24, 28, 29].

### Statistical analysis

Preliminary analyses involved a series of frequencies and cross-tabulations to estimate the prevalence of antenatal and postnatal depressive symptoms by study variables. This was followed by univariate logistic regression analysis to examine all potential risk factors associated with antenatal and postnatal depressive symptoms. Only those variables with *P*-value < 0.1 in univariate models were then entered into a multivariate model to estimate the factors associated with antenatal and postnatal depressive symptoms. Univariate and multivariate odds ratios (ORs) and their 95% confidence intervals were reported in the present study.

### Missing data

**Fig. 1:**
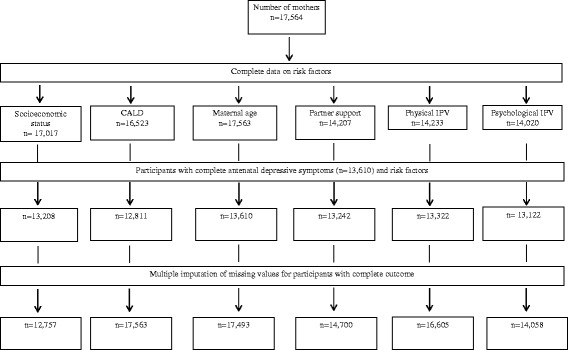
Flow chart of available data on antenatal depressive symptoms and risk factors in South Western Sydney Local Health District and Sydney Local Health District in New South Wales, Australia (2014)

**Fig. 2:**
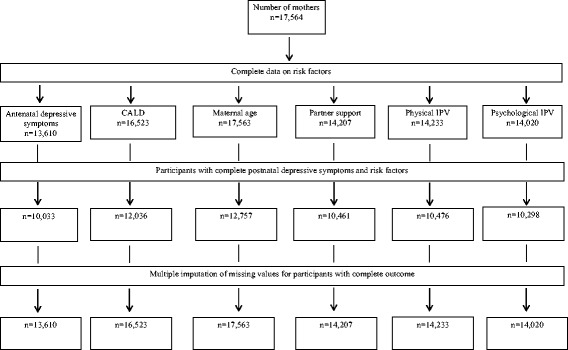
Flow chart of available data on postnatal depressive symptoms and risk factors in South Western Sydney Local Health District and Sydney Local Health District in New South Wales, Australia (2014)

The study investigated the potential effect of missing data on observed odds ratios in sensitivity analyses, which were conducted on an imputed data set based on the original cohort comprising complete outcome data (Figs. 1 and 2). Multivariate imputation by chained equation was used to impute missing information on study factors included in the analysis. This approach assumes that data were missing at random and that the characteristics of known participants can be used to calculate the characteristics of participants with missing data [30]. Sensitivity analyses were conducted using the *ice* command in Stata software (Stata Corp, V.15.0, College Station, TX, USA), based on 25 multiple imputations [31]. Revised odds ratios and corresponding confidence intervals were estimated from the imputed dataset, using the *mim* command.

## Results

### Antenatal depressive symptoms (EPDS ≥13)

The prevalence of mothers who reported depressive symptoms during pregnancy was 6.2% in the cohort and was higher among mothers who reported psychological IPV (28%) or physical IPV (32%) [Table 1]. The prevalence of ANC depressive symptoms was higher in people from the CALD background (8.0%) compared to those from the non-CALD group (4.0%). A lack of partner support (OR = 6.1, 95% CI: 4.6–7.9, *P* < 0.001) and being from the CALD group (OR = 2.0, 95% CI: 1.6–2.4, *P* < 0.001) were associated with antenatal depressive symptoms. Intimate partner violence was associated with depressive symptoms during pregnancy compared to mothers with no history of IPV (OR = 2.6, 95% CI: 1.6–4.2, P < 0.001 for physical IPV and OR = 4.8, 95% CI: 3.0–7.8, P < 0.001 for psychological IPV). Mothers from high SES group were significantly less likely to experience depressive symptoms during pregnancy compared to mothers from low SES group (OR = 0.5, 95% CI: 0.3–0.7, P < 0.001) [Table 1].

**Table 1:** Study factors and antenatal depressive symptoms of mothers from South Western Sydney and Sydney Local Health Districts in 2014 (*N* = 17,564)

				Complete cases				Multiple imputation ^a^			
Outcome	Participants	Cases	%	Unadjusted OR (95%CI)	*P* value	Adjusted OR (95%CI)	P value	Unadjusted OR (95%CI)	P value	Adjusted OR (95%CI)	P value
Antenatal depressive symptoms	13,610	846	6.2								
Socio-economic status											
Low	5716	450	7.9	1.0		1.0		1.0		1.0	
Medium	5968	321	5.4	0.6 (0.5–0.7)	< 0.001	0.8 (0.7–1.0)	0.060	0.6 (0.5–0.7)	< 0.001	0.8 (0.7–0.9)	< 0.001
High	1524	43	2.8	0.3 (0.2–0.4)	< 0.001	0.5 (0.3–0.7)	0.001	0.3 (0.2–0.4)	< 0.001	0.5 (0.4–0.5)	< 0.001
CALD group											
No	6173	278	4.5	1.0		1.0		1.0		1.0	
Yes	6638	528	8.0	1.8 (1.5–2.1)	< 0.001	2.0 (1.6–2.4)	< 0.001	1.6 (1.6–1.7)	< 0.001	1.8 (1.7–1.9)	< 0.001
Supportive Partner											
Yes	12,805	666	5.2	1.0		1.0		1.0		1.00	
No/unsure	437	140	32.0	8.5 (6.9–10.6)	< 0.001	6.1 (4.6–7.9)	< 0.001	8.5 (8.2–8.9)	< 0.001	6.1 (5.9–6.4)	< 0.001
Antenatal health problems											
No	8573	465	5.4	1.0		1.0		1.0		1.0	
Yes	3774	273	7.2	1.3 (1.1–1.5)	< 0.001	1.2 (1.0–1.5)	0.003	1.3 (1.3–1.4)	< 0.001	1.2 (1.2–1.3)	< 0.001
Physical intimate partner violence											
No	13,140	182	5.9	1.0		1.0		1.0		1.0	
Yes	777	50	27.5	6.0 (4.3–8.4)	< 0.001	2.6 (1.6–4.2)	< 0.001	6.4 (6.1–6.8)	< 0.001	3.1 (2.9–3.4)	< 0.001
Psychological intimate partner violence											
No	12,989	768	5.9	1.0		1.0		1.0		1.0	
Yes	133	43	32.3	7.0 (5.2–11.0)	< 0.001	4.8 (3.0–7.8)	< 0.001	8.0 (7.6–8.5)	< 0.001	4.1 (3.8–4.4)	< 0.001
Maternal age group											
20–34 years	9382	571	6.1	1.0		1.0		1.0		1.0	
> 35 years	3206	207	6.5	1.0 (1.1–2.5)	0.452	1.0 (0.8–1.2)	0.624	1.0 (1.0–1.1)	< 0.001	1.1 (1.0–1.1)	< 0.001
< 20 years	243	24	9.9	1.7 (1.1–2.6)	0.017	1.4 (0.8–2.5)	0.178	1.5 (1.4–1.6)	< 0.001	1.3 (1.2–1.4)	< 0.001

Data on risk factors and antenatal depressive symptoms were collected at the first antenatal clinic visit. Multivariate model adjusted for variables with p-value < 0.1 in univariate analysis. The variables include maternal age, socioeconomic status, CALD group, supportive partner, history of physical intimate partner violence, antenatal health problems and history of fear of partner/ex-partner

*CALD* culturally and linguistically diverse

^a^ Sensitivity analyses following multiple imputations for missing values

### Postnatal depressive symptoms (EPDS ≥13)

The prevalence of postnatal depressive symptoms was 3.3%, and maternal depressive symptoms during pregnancy were strongly associated with postnatal depressive symptoms (OR = 6.7, 95%CI: 4.9–9.3, *P* < 0.001) [Table 2]. Compared to mothers from the low SES group, mothers from higher SES group were less likely to experience postnatal depressive symptoms (OR = 0.7, 95% CI: 0.5–0.9, *P* = 0.010 for middle SES group and OR = 0.5, 95% CI: 0.3–0.8, *P* = 0.025 for high SES group). The prevalence of postnatal depressive symptoms was higher in mothers from the CALD group (4.2%), and mothers who identified as being from the CALD group were also more likely to experience postnatal depressive symptoms compared to those, not from the CALD group (OR = 1.5, 95%CI 1.1–2.0, *P* = 0.002). Psychological IPV was strongly associated with postnatal depressive symptoms (OR = 5.3, 95% CI: 2.5–11.0, *P* < 0.001), and mothers who had a caesarean section were more likely to experience postnatal depressive symptoms compared to those who had normal vaginal delivery (OR = 1.7, 95%CI: 1.3–2.3, P < 0.001).

**Table 2:** Study factors and postnatal depressive symptoms of mothers from South Western Sydney and Sydney Local Health Districts in 2014 (N = 17,564)

				Complete cases				Multiple imputation ^a^			
Outcome	Participants	cases	%	Unadjusted OR (95%CI)	P value	Adjusted OR (95%CI)	P value	Unadjusted OR (95%CI)	P value	Adjusted OR (95%CI)	P value
Postnatal depressive symptoms	12,757	425	3.3								
Maternal age group											
20–34 years	8605	291	3.4	1.0		1.0		1.0		1.0	
> 35 years	3170	101	3.2	0.9 (0.7–1.1)	0.638	0.8 (0.6–1.2)	0.422	0.9 (0.9–1.0)	0.002	0.7 (0.8–0.9)	< 0.001
< 20 years	228	8	3.5	1.0 (0.5–2.1)	0.936	1.3 (0.5–3.)	0.525	1.1 (0.9–1.2)	0.094	0.9 (0.8–1.1)	0.862
Socio-economic status											
Low	4964	211	4.3	1.0		1.0		1.00		1.0	
Medium	5877	162	2.8	0.6 (0.5–0.7)	< 0.001	0.7 (0.5–0.9)	0.010	0.6 (0.6–0.7)	< 0.001	0.7 (0.7–0.8)	< 0.001
High	1559	36	2.3	0.5 (0.3–0.7)	0.001	0.5 (0.3–0.8)	0.025	0.5 (0.4–0.5)	< 0.001	0.5 (0.5–0.6)	< 0.001
Antenatal depressive symptoms											
EPDS < 13	9405	225	2.4	1.0		1.0		1.00		1.0	
EPDS ≥13	628	97	15.5	8.5 (6.5–11.0)	< 0.001	6.7 (4.9–9.3)	< 0.001	8.5 (8.2–8.8)	< 0.001	6.9 (6.6–7.2)	< 0.001
CALD group											
No	6031	162	2.7	1.0		1.0		1.0		1.0	
Yes	6005	252	4.2	1.6 (1.3–1.9)	< 0.001	1.5 (1.1–2.0)	0.002	1.4 (1.4–1.5)	< 0.001	1.3 (1.2–1.4)	< 0.001
Supportive Partner											
Yes	10,131	299	3.0	1.0		1.0		1.0		1.00	
No/unsure	103	36	10.9	1.0 (0.6–1.6)	0.973	3.1 (2.0–4.9)	< 0.001	3.8 (3.6–4.0)	< 0.001	2.9 (2.7–3.2)	< 0.001
Antenatal health problems											
No	8566	256	3.0	1.0		1.0		1.0		1.0	
Yes	3177	123	3.9	1.3 (1.0–1.6)	0.400	1.1 (0.8–1.4)	0.287	1.3 (1.2–1.3)	< 0.001	1.2 (1.2–1.3)	< 0.001
Physical intimate partner violence											
No	10,339	329	3.1	1.0		1.0		1.0		1.0	
Yes	137	11	8.0	2.8 (1.5–5.3)	0.001	0.9 (0.3–2.3)	0.864	3.2 (2.9–3.5)	< 0.001	1.5 (1.3–1.7)	< 0.001
Psychological intimate partner violence											
No	10,196	317	3.1	1.0		1.0		1.0		1.0	
Yes	102	15	14.7	5.5 (3.1–9.6)	< 0.001	5.3 (2.5–11.0)	< 0.001	5.7 (5.3–6.2)	< 0.001	4.6 (4.1–5.1)	< 0.001
Type of delivery ^**b**^											
Normal vaginal	7865	219	2.8	1.0		1.0		1.0		1.0	
Assisted vaginal	1408	55	3.9	1.4 (1.0–1.9)	0.014	1.3 (0.8–2.0)	0.166	1.2 (1.2–1.3)	< 0.001	1.3 (1.2–1.4)	< 0.001
Caesarean section	3448	151	4.4	1.6 (1.3–2.0)	< 0.001	1.7 (1.3–2.3)	< 0.001	1.4 (1.4–1.5)	< 0.001	1.4 (1.3–1.5)	< 0.001

Information on maternal age, supportive partner, physical and psychological intimate partner violence, socio-economic status, antenatal depressive symptoms, CALD and antenatal health problems were collected during the antenatal period

*CALD* culturally and linguistically diverse

^a^ Sensitivity analyses following multiple imputations for missing values

^b^ Information on the type of delivery was collected soon after birth; and data on postnatal depressive symptoms were collected within the first six weeks post-birth. Multivariate model adjusted for variables with p-value < 0.1 in univariate analysis. The variables include maternal age, antenatal depressive symptoms, socioeconomic status, CALD, supportive partner, history of physical intimate partner violence, antenatal health problems, history of fear of partner/ex-partner and type of delivery

Stratified analyses showed no substantial, significant differences between CALD and non-CALD groups. For example, mothers who experienced physical and psychological IPV were almost equally distributed in both groups (50% in both groups for physical IPV, and 49.0% and 51.0% among non-CALD and CALD population for psychosocial IPV, respectively). The revised odds ratios from sensitivity analyses were not markedly different from the complete case analysis (except for the association between younger maternal age and antenatal depressive symptoms), indicating that missing data did not substantially affect the observed findings.

## Discussion

The prevalence of maternal depressive symptoms during pregnancy was 6.2%, with a higher prevalence observed in mothers with a history of IPV. The analysis showed that a lack of partner support, IPV, being from the CALD population and low SES group were associated with maternal depressive symptoms during pregnancy. The prevalence of postnatal depression was 3.3%, and the risk factors for postnatal depressive symptoms were assisted delivery (caesarean section), low SES, psychological IPV, being from the CALD population and antenatal depressive symptoms.

The impact of antenatal depression on perinatal outcomes in the study participants has been documented [10] and was consistent with previous reports [5, 11]. Similarly, most of the previously established risk factors for antenatal depression in other contexts also played a role in predicting maternal depressive symptoms during pregnancy in the current study. A number of the identified risk factors are amenable to initiatives, suggesting that well-targeted interventions and policies may be helpful in reducing the burden of antenatal depression. For example, IPV and a lack of partner support were the strongest risk factors for antenatal depressive symptoms in the present study, consistent with evidence from previous reports [12, 32]. The Australian, State and Territory governments commitment to reducing perinatal depression, IPV and improve partner support are broader initiatives needed to improve the social-emotional and physical health of both mother and infant health [33–35].

In the state of NSW, there are facility- and community-based interventions that aim to identify vulnerable mothers with probable depressive episodes quickly, and referral for expert assessment and subsequent management [34, 36–38]. While those initiatives are important and reportedly well-received, it is also worth noting that risk factors for antenatal depression are multi-faceted, and would require multidisciplinary and multi-agency intervention approaches that are targeted, collaborative and coordinated to improve health outcomes [39]. More recently, the NSW government has begun moving to a more patient-centred integrated health system, with connected service provision across different providers [40]. Aspects of the strategy have been shown to identify barriers to care and facilitate care for vulnerable families, including those with depressive symptoms [41, 42]. Scaling up such an approach for at-risk mothers will improve not only maternal and infant health, but also the family’s social interaction and long-term productivity.

The study found that the prevalence of antenatal depressive symptoms was similar to a previous national report [4]. This finding underscores the utility of the EPDS, applied in the Australian primary health care setting, to promptly identify women with probable depression and referral for professional assessment. Our study indicated that the proportion of women who reported postnatal depressive symptoms was lower than the national prevalence (19%) [43] and prior studies from Australia (6.2–10.4%) [11, 13, 44]. However, a recent systemic review found that depression among Australian women ranged from 2.6% to 43.9%, highlighting the disparities in mental health burden across the lifespan [43, 45].

A plausible explanation for the low prevalence of postnatal depressive symptoms in our study may be due to the period in which the data were collected. The postnatal data were collected within the first six weeks’ post-birth, while the national survey was based on the first year of the birth of the child. Additionally, the prevalence of postnatal depressive symptoms may have varied from previous studies because of the impact of the integrated perinatal care initiatives [36, 46] and other family-centred services in the region [47]. Formal evaluation of the perinatal care interventions is warranted to determine the extent to which the initiatives have been context-specific.

Mothers from low SES groups and being from the CALD group were at risk of experiencing pre- and/or post-pregnancy depressive symptoms, in line with previous studies [12, 14]. Plausible reasons for why being from the CALD group increases the risk of prenatal and/or postnatal depressive symptoms among mothers have been highlighted in Australia. These include socio-cultural barriers (e.g., cultural norms, language and traditional gender roles); structural barriers (e.g., a lack of knowledge of available services and issues with access); service-related barriers (e.g., culturally-inappropriate service models or a perception of such services); domestic violence problems [48], and alcohol and other drugs issues [49]. A number of those barriers may also apply to mothers from low SES groups, as well as issues of limited uptake of health messages associated with mothers from low-income households [50]. Although being from the CALD group is not modifiable or improvement in SES status can be a long-term project; targeted and culturally-appropriate initiatives that are specific to mothers in those populations will improve health outcomes. A report from Australia estimated that maternal perinatal depression cost the Australian economy 171 million dollars in 2012, and the majority of the burden was attributable to lost productivity in the workplace [35]. This report further highlights the fact that routine screening for perinatal depression and subsequent referral for further assessment of vulnerable women is essential to improving health and economic productivity.

The study has a number of policy and pragmatic implications for health professionals, health administrators, researchers and the public. Our study provides risk factors for antenatal and postnatal depressive symptoms in Australia, as well as among people from culturally diverse communities, using routinely collected and context-specific data. Evidence has shown that strong government support is critical to health interventions aimed at identifying and/or treating diseases in the early stages [4, 16]. The NSW government SAFE START policy framework is one of such support, aimed at providing planned, coordinated and complete psychosocial assessment, depression screening and follow-up care and support for mothers and babies during the perinatal period [36]. Although the SAFE START initiative is still active in NSW, in 2015, the Australian federal government stopped a funding agreement with the states and territories government for a National Perinatal Depression Initiative (NPDI) which provided new parents access to screening and counselling services since the year 2008 [51]. Studies that evaluate the impact of the NPDI and other sub-national policy responses and interventions to perinatal depression may be warranted in Australia to measure where improvements have been made, where opportunities are or where challenges occur. Further studies on the impact and possible referral pathways of moderate perinatal depression may also be needed.

### Study limitations and strengths

The study has a number of limitations. First, the outcome of interest and some of the risk factors (e.g., IPV) were based on self-report, which is a possible source of recall or measurement bias that may have underestimated or overestimated the association between the risk factors and outcomes. Second, the study used routinely collected data for the analyses of risk factors for antenatal depressive symptoms, and the establishment of clear temporal relationships cannot be determined for such associations, where study factors data and antenatal depressive information were collected contemporaneously. Nonetheless, the study provides evidence on clear temporal relationships between the study factors and postnatal depressive symptoms. Third, unmeasured covariates such as the impact of social support and family structure may be a limitation of the study. Fourth, our study was unable to differentiate mothers with pre-existing clinical depression from those with first-ever perinatal depressive symptoms. A stratified analysis of pre-existing and first-ever perinatal depression would have provided additional information on the health status of mothers. Despite these limitations, we take advantage of the routine and consistently collected maternal and infant data in Sydney, Australia to provide context-specific evidence to inform policy decision-making and initiatives. Also, the observed odds ratios are unlikely to be affected by missing data as we considered the potential bias due to missing data in a sensitivity analysis that imputed missing information.

The EPDS is a screening tool, and may not identify all mothers with depressive episodes, as some mothers with high scores may not have clinical depression, while some mothers with low scores may underestimate, or not wish to report distress. The use of the EPDS as the measure for probable depression diagnosis may be less ideal as an assessment of depressive symptoms in the postnatal period may due to extra postpartum duties associated with the arrival of a newborn. Possible limitations of the EPDS tool and an alternative device were recently highlighted [52], similar to previously published shortcomings [53, 54] and alternatives to the EPDS [55, 56]. Nevertheless, it is important to note that even if a screening tool is error-free, its interpretation (i.e., the assignment of a positive or negative outcome) may still be incorrect [57].

Despite the limitations of the EPDS, it is an extremely valuable tool for researchers to evaluate the impact of preventive programs and for clinicians to promptly identify mothers who may be experiencing or are at risk for perinatal depression [4, 16]. Also, the EPDS is unique and designed for use in the perinatal period as it does not measure somatic symptoms associated with depression, as symptoms like weight change and sleep difficulties are associated with normal pregnancies.

## Conclusion

The prevalence of antenatal depressive symptoms was higher in mothers with a history of IPV. A lack of partner support, IPV, being from the CALD population and low SES group were risk factors for antenatal and postnatal depressive symptoms. Antenatal depressive symptoms were strongly associated with postnatal depressive symptoms. The study suggests that screening for probable depression and referral for expert assessment may help to identify at-risk mothers of perinatal depression promptly, and may create pathways for improved maternal mental health care.

## Abbreviations

CALD: Culturally and linguistically diverse
EPDS: Edinburgh postnatal depression scale
IPV: Intimate partner violence
NPDI: National perinatal depression initiative
NSW: New South Wales
SES: Socio-economic status
SLHD: Sydney Local Health District
SWSLHD: South Western Sydney Local Health District
